# Epidemiology of SARS-CoV-2, Influenza, RSV, and Human Metapneumovirus Across the COVID-19 Era: An Eight-Year Tertiary-Center Study in Saudi Arabia

**DOI:** 10.3390/pathogens15090918

**Published:** 2026-08-31

**Authors:** Lama Alzamil, Arwa Bagasi, Zeina S. Alkudmani, Hamad M. Almatrudi, Abdulrahman F. Alrezaihi, Abdulaziz M. Almuqrin

**Affiliations:** Department of Clinical Laboratory Sciences, College of Applied Medical Sciences, King Saud University, Riyadh 12372, Saudi Arabia

**Keywords:** respiratory pathogens, COVID-19, SARS-CoV-2, influenza, respiratory syncytial virus, human metapneumovirus, Saudi Arabia

## Abstract

Background: Respiratory tract infections (RTIs) caused by viruses are a public health problem, contributing to hospital admissions and morbidity worldwide. The COVID-19 pandemic substantially altered the detection patterns of respiratory viruses through public health interventions. Longitudinal data on these changes and viral seasonality remain limited in Saudi Arabia. This eight-year study evaluated epidemiological and seasonal patterns of major respiratory viruses across the COVID-19 era at a major Saudi tertiary care center. Methods: A retrospective observational study was conducted at King Khalid University Hospital, Riyadh, from October 2017 to December 2025. PCR-confirmed detections for SARS-CoV-2, Influenza A, Influenza B, respiratory syncytial virus (RSV), and human metapneumovirus (HMPV) were extracted from the hospital Laboratory Information System. Demographics, co-detection, seasonality, and length of stay (LOS) were analyzed across four eras spanning the pre-COVID-19, lockdown, restriction, and post-restriction periods using descriptive statistics, the Kruskal–Wallis test, and multivariable negative binomial regression. Results: Among 5708 confirmed respiratory viral detection episodes, SARS-CoV-2 was the most frequently detected (52.5%), followed by Influenza A (19.1%), RSV (16.4%), Influenza B (8.4%), and HMPV (3.6%). Before the pandemic, Influenza A accounted for the largest proportion of detected episodes (71.1%), with detections clustering particularly during autumn and winter seasons, whereas SARS-CoV-2 accounted for the majority of detected episodes during the lockdown and restriction eras (97.4% and 93.2%). The post-restriction era became more diverse, with RSV being detected in substantial proportions (28.3%), showing prominent winter activity. RSV and HMPV predominantly affected children aged 0–5 years, whereas SARS-CoV-2 was more frequently detected among older adults. Among adults, older age was associated with prolonged LOS; Influenza A and HMPV were associated with shorter stays compared to SARS-CoV-2. Among children, no virus was independently associated with LOS after adjustment for age and sex; however, older age was associated with longer hospital stays. Conclusion: The observed detection patterns of major respiratory viruses shifted substantially across the COVID-19 eras, with notable post-restriction diversification among observed positive detections. LOS was associated with age in both adults and children and with virus type among adults, supporting continued surveillance to guide preventive and clinical strategies.

## 1. Introduction

Respiratory tract infections (RTIs) are a major cause of clinical burden worldwide, contributing to hospital admissions, morbidity, and millions of deaths each year, particularly in developing countries [1,2]. They affect individuals across all age groups, specifically young children, older adults, and people with chronic medical conditions [3,4].

RTIs are caused by a wide range of viral pathogens. During the COVID-19 pandemic, SARS-CoV-2 became a major cause of respiratory infections and substantially altered the detection patterns of other respiratory viruses in many countries. These changes were largely attributed to public health interventions, including travel restrictions, lockdowns, mask use, school closures, and reduced social contact [5,6,7,8]. Nevertheless, other respiratory viruses, including influenza A, influenza B, respiratory syncytial virus (RSV), and human metapneumovirus (HMPV), remained important causes of RTIs, particularly after the easing of COVID-19 restrictions.

Saudi Arabia is an important setting for respiratory virus surveillance because of its large population, international connectivity, and role in hosting major religious gatherings, such as Hajj and Umrah, which attract millions of pilgrims from around the world each year [9]. In addition, the country has a large expatriate workforce, including nearly 12 million foreign workers from more than 100 countries, many of whom travel regularly between Saudi Arabia and their countries of origin [10]. These factors may influence the introduction, circulation, and spread of respiratory viral infections; for example, the post-restriction increase in measles cases in the country has been linked in part to importation pressure from mass gatherings and to susceptibility gaps within its large non-national population [11]. Together, these dynamics underscore the need for continued molecular surveillance to monitor changes in RTI epidemiology, as recently emphasized for endemic seasonal coronaviruses circulating in Riyadh [12].

Respiratory virus circulation and seasonality vary by geographic region, with seasonal peaks influenced by different factors such as climate, population behavior, and public health measures. Following the COVID-19 pandemic, several countries reported changes in the timing and magnitude of seasonal respiratory virus activity [13]. To date, however, most Saudi studies spanning the COVID-19 era have focused on single pathogens or limited time periods, such as the molecular characterization of HMPV among hospitalized children during the pandemic [14], and longitudinal data examining the seasonality and epidemiological changes in multiple respiratory viruses across the COVID-19 era remain limited in the country.

Therefore, this eight-year study at a major Saudi tertiary healthcare center aimed to evaluate changes in the epidemiological and seasonal patterns of major respiratory viruses across the COVID-19 era. The analysis included SARS-CoV-2, influenza A, influenza B, RSV, and HMPV. Age distribution, co-detection patterns, presenting symptoms, comorbidities, and length of hospital stay were also assessed to provide a broader overview of the clinical and epidemiological burden of these viruses in this setting.

## 2. Materials and Methods

### 2.1. Study Design, Data Source, and Laboratory Testing

Following approval from the Institutional Review Board of King Saud University (IRB E-25-10534), this retrospective single-center study was conducted at King Khalid University Hospital (KKUH), a tertiary academic center in Riyadh, Saudi Arabia, over an approximately eight-year period (October 2017–December 2025). Data were extracted electronically from the Oracle Health (Cerner) electronic health record (EHR) using predefined structured microbiology, demographic, comorbidity, and encounter fields; no manual extraction was performed, and the analytical dataset was de-identified before analysis. Given the retrospective use of de-identified data, individual informed consent was waived.

All PCR-confirmed positive detections for SARS-CoV-2, Influenza A, Influenza B, RSV, and HMPV during the study period were included; negative, invalid, pending, and non-respiratory results were excluded. Viral pathogens were detected from nasopharyngeal aspirates using the BioFire^®^ FilmArray^®^ multiplex PCR system (BioFire Diagnostics, LLC., Salt Lake City, UT, USA). During 2017–2020, the Respiratory Panel 2 (RP2) was in routine use and detected Influenza A, Influenza B, RSV, and HMPV. In February 2020, the laboratory transitioned to Respiratory Panel 2.1 (RP2.1), which retained all prior targets and added SARS-CoV-2.

### 2.2. Variable Definitions and Classifications

The unit of analysis was the virus-specific detection episode, defined as a unique positive detection of a specific virus. To define the clinical episode operationally, we used a 14-day window: positive detections for any virus occurring within 14 days of the first detection in that patient were considered part of the same clinical episode; detections occurring ≥14 days apart were counted as separate clinical episodes, even if within the same patient. Co-detection was defined as the detection of two or more target viruses within the same 14-day clinical episode, with each virus contributing a separate virus-specific detection episode.

The total dataset comprises 5708 virus-specific detection episodes, representing 5648 unique clinical episodes across 5645 unique patients. Because patients may experience distinct episodes over time, these three quantities are reported separately throughout the manuscript. Length of stay (LOS) was the number of midnights between admission and discharge (EHR “LOS (Midnight)” variable); for the sensitivity analysis, prolonged LOS was defined as >7 nights. The care setting was classified as inpatient or non-inpatient (emergency, outpatient, day care, or telehealth). Seasons were winter (December–February), spring (March–May), summer (June–August), and autumn (September–November). To ensure complete, uniformly distributed seasons, December detections were assigned to the following year’s winter (e.g., December 2021 was included in Winter 2022). Additionally, 184 episodes were excluded from this seasonal analysis to ensure complete seasons (96 from October–November 2017; 88 from December 2025). Based on Saudi COVID-19 policy, four eras were defined: Pre-COVID (October 2017–22 March 2020), Lockdown (23 March–20 June 2020), Restriction (21 June 2020–31 May 2022), and Post-restriction (1 June 2022–December 2025).

Age groups were mutually exclusive across all analyses. Descriptive tables used 0–5, 6–17 (<18 years, pediatric), 18–40, 41–59, and ≥60 years; the adult regression used 18–40 (reference), 41–59, and ≥60 years, and the pediatric regression used 0–5 (reference), 6–11, and 12–17 years. The <18/≥18 boundary gave no overlap or gap at age 18.

Comorbidities, clinical diagnoses (pneumonia, bronchiolitis), and presenting symptoms (fever, cough, shortness of breath) were taken from structured EHR fields. Comorbidities were flagged as binary present/absent variables from the “All_Complications” field by keyword search: Diabetes mellitus, systemic hypertension (excluding pulmonary hypertension), heart failure, asthma, and pulmonary hypertension. These variables were recorded by treating clinicians during routine care and were not independently adjudicated; categories were not mutually exclusive, and missing entries for symptoms/diagnoses were not systematically documented.

### 2.3. Statistical Analysis

Analyses used Python 3.14.6 (pandas 3.0.3, NumPy 2.5.0, SciPy 1.18.0, StatsModels 0.14.4). Categorical variables are summarized as *n* (%) and continuous variables as medians (IQR). For co-detection analyses, each co-detection event was counted once, while each detected virus contributed separately to its virus-specific descriptive denominator. Co-detected episodes were excluded from the LOS regression analyses to preserve the single-virus independence assumption.

LOS analyses were restricted to inpatient episodes with complete LOS and one target virus, stratified into adults (≥18 years, *n* = 2909) and children (<18 years, *n* = 1435); together these represent 4344 of 4518 inpatient episodes. To achieve these cohorts, 102 co-detected virus-specific inpatient episodes (arising from 51 inpatient co-detection events, of the 60 co-detection events overall) were first excluded. Subsequently, 53 adult episodes and 19 pediatric episodes were excluded for missing length-of-stay data or missing covariates. The same patient could contribute multiple episodes if readmitted. The study flow is summarized in Figure S1. The 2909 analyzed adult episodes were contributed by 2908 unique patients, and the 1435 pediatric episodes were contributed by 1434 unique patients.

For adults, the primary model was negative binomial regression of continuous LOS (nights), reporting incidence rate ratios (IRR); overdispersion was present (α ≈ 0.68). A sensitivity analysis used modified Poisson regression with patient-clustered robust standard errors for prolonged stay (>7 nights), reporting risk ratios (RR). Both were adjusted for age group, sex, pathogen, and the five comorbidities. For children, LOS was compared across pathogens by Kruskal–Wallis test with Dunn’s post hoc test and Bonferroni correction (10 comparisons); a multivariable negative binomial model adjusted for pathogen, age group (0–5, 6–11, 12–17), and sex. To account for repeated episodes, patient-clustered robust standard errors were used for all LOS regression models. Furthermore, a sensitivity analysis restricted to one randomly selected episode per patient (*n* = 2908 adults; *n* = 1434 children) yielded materially identical results. Estimates are reported with 95% CIs; *p* < 0.05 was considered significant. To further address the strong structural association between virus type and calendar era, a sensitivity analysis restricted to the post-restriction era (where all five viruses were co-circulating) was performed for the adult cohort.

## 3. Results

### 3.1. Demography and Epidemiology

**Figure 1:**
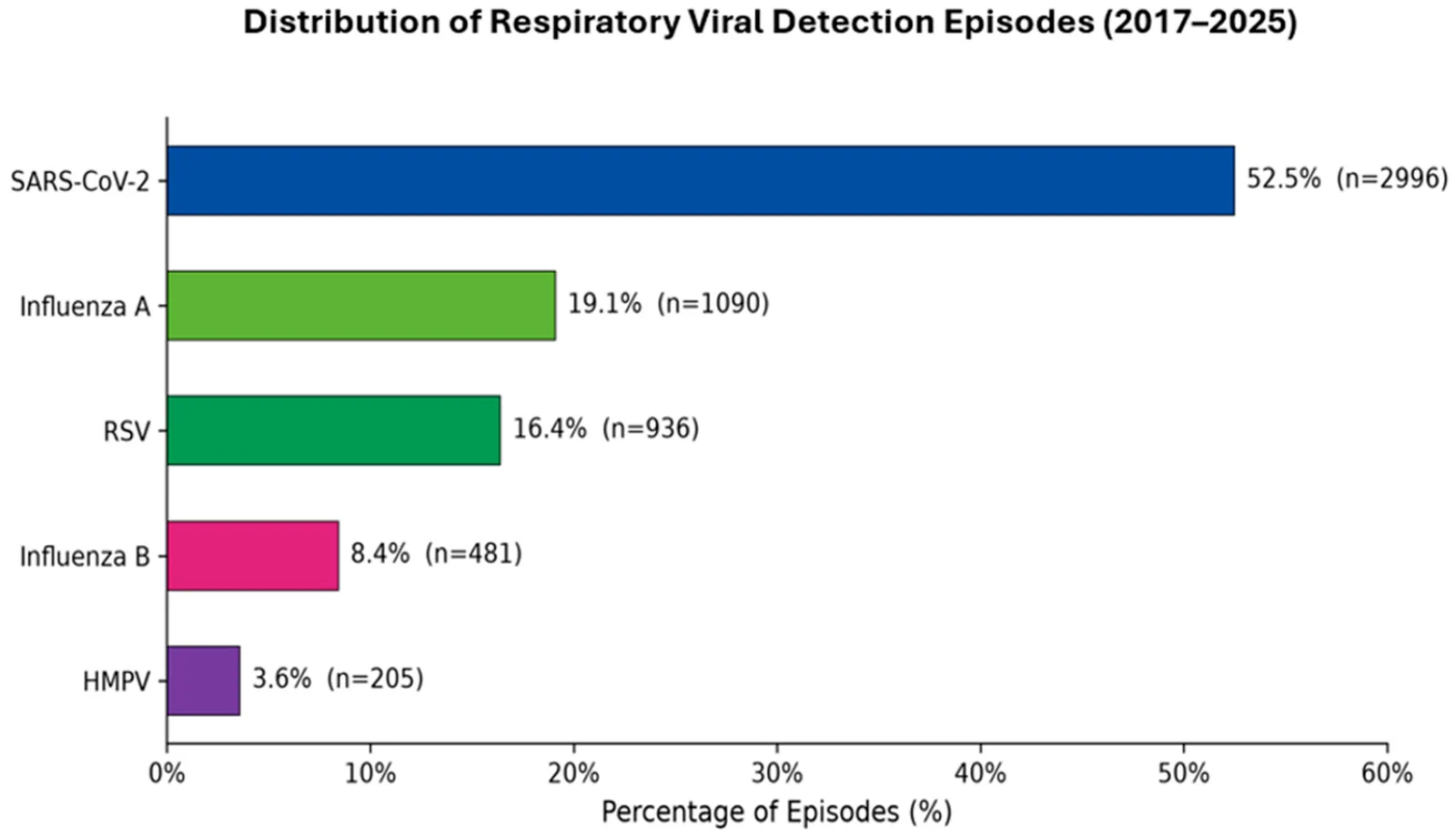
Distribution of confirmed respiratory viral detection during the study period. Bars show the proportion and number of each virus among 5708 confirmed viral detection episodes, including SARS-CoV-2, influenza A, RSV, influenza B, and HMPV.

As illustrated in Figure 1, 5708 confirmed respiratory viral episodes were identified during the study period. SARS-CoV-2 was the most frequently identified pathogen (*n* = 2996, 52.5%), followed by Influenza A (*n* = 1090, 19.1%), RSV (*n* = 936, 16.4%), Influenza B (*n* = 481, 8.4%), and HMPV (*n* = 205, 3.6%).

The demographic characteristics of the confirmed respiratory viral detection episodes are detailed in Table 1. The sex distribution was approximately equal, with 2925 females and 2783 males. Adults aged ≥60 years represented the largest age group (28.2%), followed by children aged 0–5 years (26.3%). Saudi nationals comprised most confirmed episodes (85.7%), while non-Saudi individuals accounted for 819 episodes (14.3%). Overall, 79.2% of episodes were recorded in the inpatient setting and 20.4% in non-inpatient settings, while the care setting was unknown for 23 episodes (0.4%).

### 3.2. Co-Detection Analysis

Co-detection analysis identified 60 co-detection events, representing approximately 1.1% of all unique clinical episodes (60 of 5648). These 60 events generated 120 virus-specific detections, representing 2.1% of the total 5708 detection episodes. RSV was the most commonly co-detected virus. The majority of co-detected cases were reported in patients aged <18 years, particularly those aged 0–5 years (61.7%) and 6–17 years (20.0%) (Table 2).

### 3.3. Virus-Specific Demographic Characteristics

Virus-specific demographic characteristics are summarized in Table 3. RSV and HMPV were predominantly detected among young children, with 71.4% and 51.7% of episodes, respectively, occurring in patients aged 0–5 years. In contrast, SARS-CoV-2 was most frequent among older adults, with 36.5% of episodes in subjects aged ≥60 years. Influenza A demonstrated a broad age distribution, with substantial proportions across all age groups. Influenza B was more commonly detected in children and younger adults, with 27.7% and 27.4% of episodes in the 0–5 and 6–17 years’ age groups, respectively.

### 3.4. Changes in Respiratory Virus Distribution Across COVID-19 Eras

**Figure 2:**
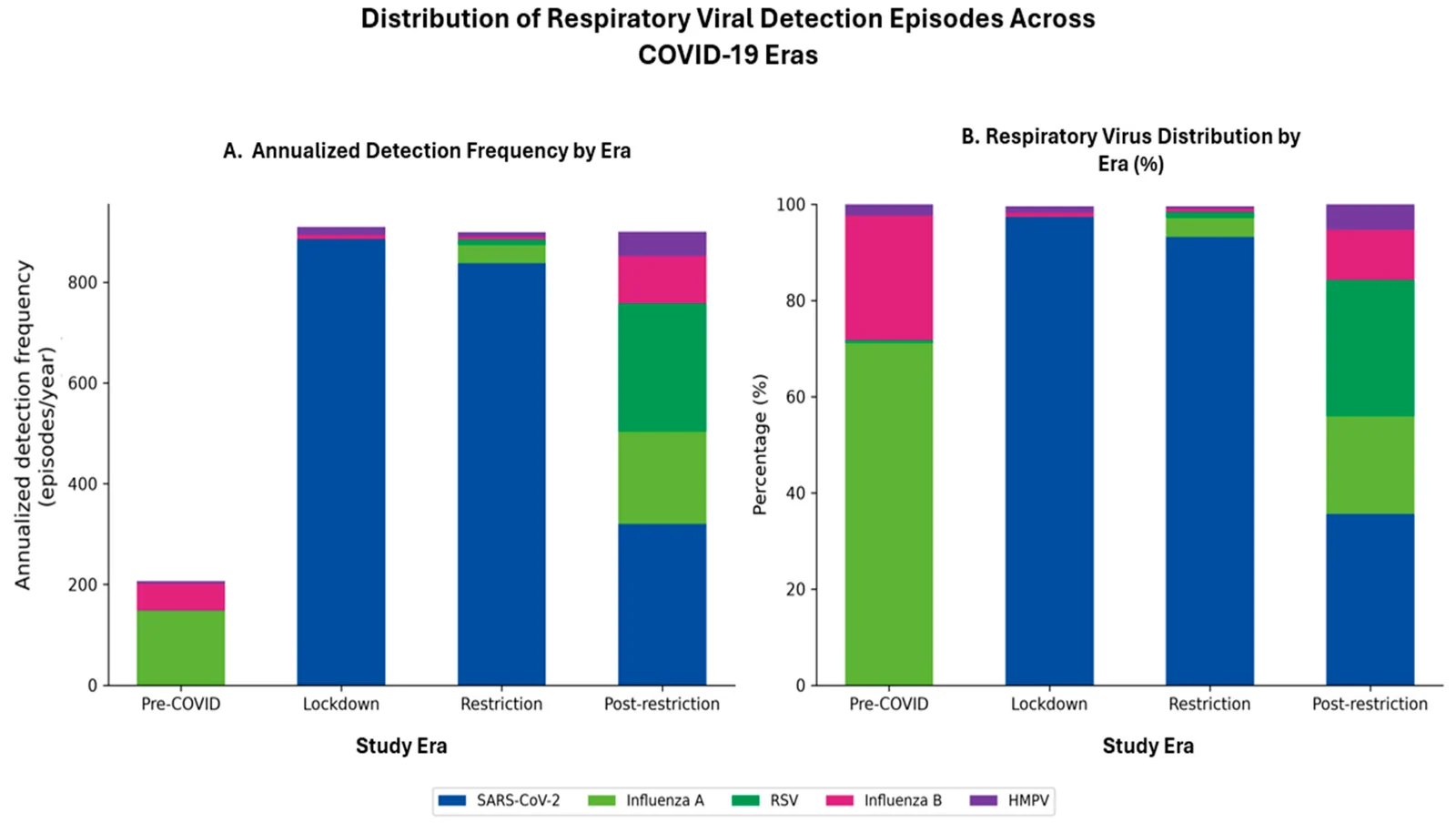
Distribution of confirmed respiratory viral detection episodes across the COVID-19 eras. Annualized detection frequencies (episodes per year, normalized to each era’s duration) are shown in (**A**). The percentage distribution of confirmed respiratory pathogens across the COVID-19 eras is illustrated in (**B**). Frequencies and proportions reflect detected episodes and are not adjusted for testing volume.

The distribution of respiratory viruses varied markedly across the four COVID-19 eras (Figure 2; Table 4). To account for the different durations of the eras, annualized detection frequencies (episodes per year, normalized to each era’s duration) were calculated and are reported alongside percentages. These reflect observed positive detections and are not adjusted for testing volume.

In the pre-COVID era (29.6 months), Influenza A was the most frequently detected virus, accounting for 71.1% of observed detections (annualized detection frequency 146.9 per year), followed by Influenza B (25.9%; 53.6 per year). RSV (0.6%; 1.2 per year) and HMPV (2.4%; 4.9 per year) were detected only rarely during this period.

As expected, SARS-CoV-2 rapidly became predominant during the lockdown era (3.0 months), representing 97.4% of all detected respiratory viruses (annualized detection frequency 885.4 per year). Notably, no Influenza A or RSV detections were reported during this period, while Influenza B (0.9%; 7.9 per year) and HMPV (1.7%; 15.9 per year) accounted for the remaining detections.

During the restriction era (23.3 months), SARS-CoV-2 remained the most frequently detected virus (93.2%; annualized detection frequency 837.7 per year), followed distantly by Influenza A (4.1%; 36.6 per year), RSV (1.1%; 10.3 per year), HMPV (1.0%; 8.8 per year), and Influenza B (0.6%; 5.7 per year).

In the post-restriction era (43.0 months), the distribution of detected respiratory viruses became more diverse. While SARS-CoV-2 remained the most frequently detected virus (35.6%; annualized detection frequency 320.1 per year), RSV emerged as a major respiratory pathogen (28.3%; 254.8 per year), followed by Influenza A (20.4%; 183.3 per year), Influenza B (10.4%; 93.8 per year), and HMPV (5.3%; 48.0 per year). All non-SARS-CoV-2 viruses showed higher annualized detection frequencies than during the restriction era.

This shift in the distribution of detected respiratory viruses was accompanied by a marked demographic transition toward younger age groups (Table 4). Children aged 0–5 years accounted for 39.3% of respiratory infections in the post-restriction era (compared with 8.2% during the restriction era), while the proportion of patients aged ≥60 years declined from 38.6% to 21.4%.

### 3.5. Seasonal Distribution of Respiratory Viruses

**Figure 3:**
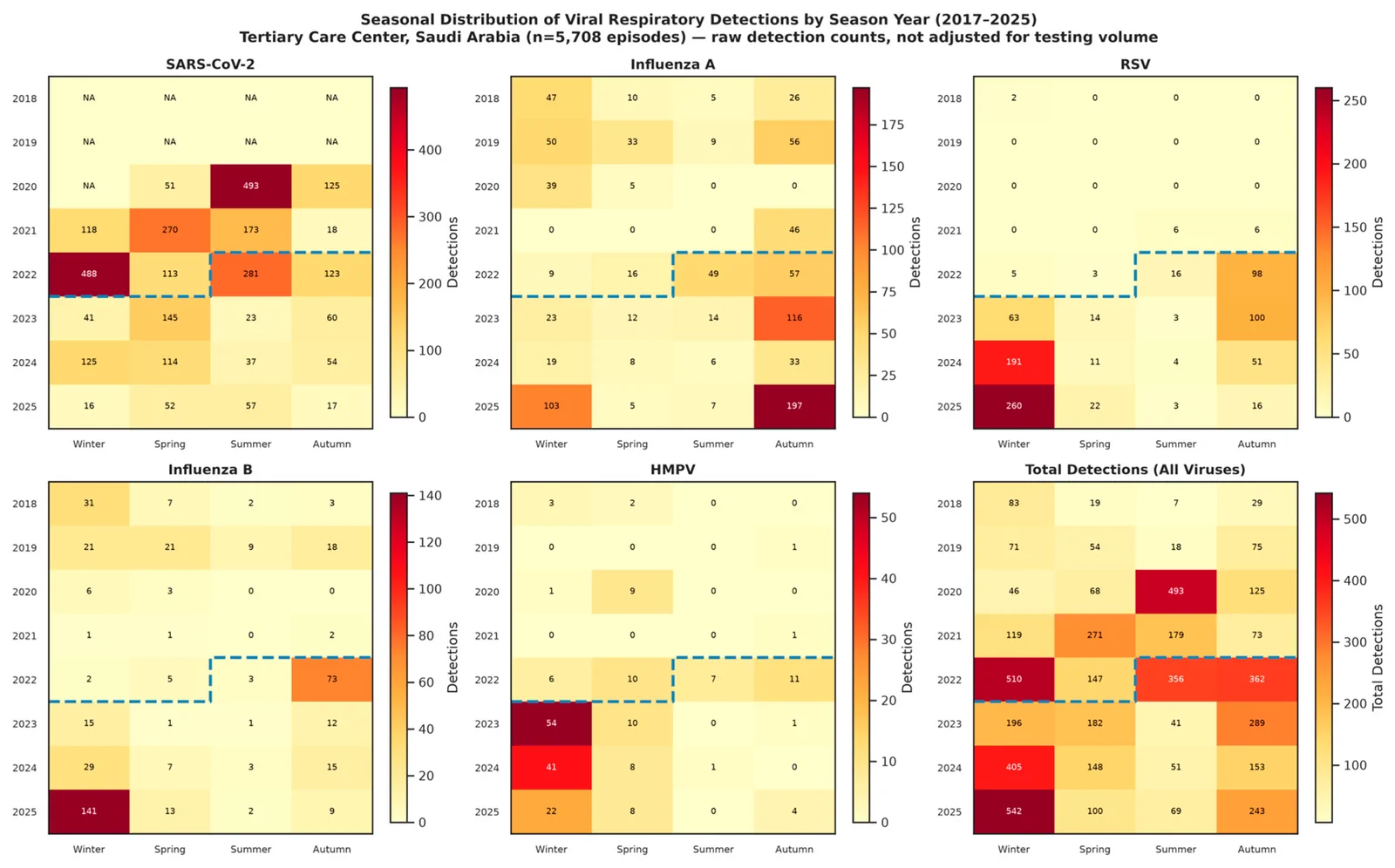
Seasonal distribution of respiratory viral detections by year (2017–2025). Heatmaps show the raw seasonal counts of detected episodes for SARS-CoV-2, influenza A, RSV, influenza B, and HMPV, alongside total detections across all viruses (**far right**). Seasons were defined epidemiologically as winter (December–February), spring (March–May), summer (June–August), and autumn (September–November); December was assigned to the following year’s winter. To ensure complete seasons, 184 episodes were excluded (96 from October–November 2017; 88 from December 2025). White cells marked “NA” for SARS-CoV-2 indicate no detection test was available. Color scales are independently normalized per virus. Counts are not adjusted for testing volume. The dashed stepped line indicates the transition to the post-restriction period (June 2022); it steps between the spring and summer 2022 columns because, under the epidemiological-season definition, winter and spring 2022 fall within the restriction era whereas summer 2022 onward are post-restriction.

As illustrated in Figure 3, seasonal patterns of the study viruses were analyzed across the COVID-19 eras. Our observations showed clear seasonal activity of Influenza A and Influenza B during autumn and winter, while RSV and HMPV cases were rarely detected before the COVID-19 pandemic. During the Lockdown and Restriction eras, SARS-CoV-2 dominated the seasonal distribution, with major peaks in summer 2020 and winter 2022 (493 and 488 detections, respectively), while most non-SARS-CoV-2 viral respiratory viruses were markedly reduced.

Following the easing of restrictions, the seasonal activity of viral respiratory pathogens became diverse. Influenza A was detected at higher levels with prominent autumn peaks, particularly in 2023 and 2025. Influenza B increased mainly during winter, especially in 2024 and 2025. RSV showed increases in detection, with strong winter activity from 2023 onwards and the highest winter peaks in 2024 and 2025. Similarly, HMPV detections clustered mainly in winter (Figure 3).

### 3.6. Comorbidities and Presenting Symptoms

Comorbidities and presenting symptoms of the confirmed respiratory viral detection episodes are reported in Table 5. Fever, pneumonia, shortness of breath, and cough were the most frequently documented clinical findings, accounting for 32.7%, 19.3%, 15.6%, and 13.0%, respectively; viral pneumonia or community-acquired pneumonia (CAP) specifically accounted for 6.9% of cases. In addition, diabetes mellitus represented the most common chronic pre-existing comorbidity (13.2%), followed by hypertension (8.9%) and asthma (6.8%).

Virus-specific analysis showed that fever was reported in half of HMPV, RSV, and Influenza B episodes. Influenza A showed the highest frequency of pneumonia of any type (23.6%), followed by HMPV (22.4%), whereas viral pneumonia/CAP specifically was most frequent among SARS-CoV-2 episodes (8.9%). Notably, bronchiolitis was reported in 24.0% of RSV episodes, which was substantially higher than in other viruses. Asthma was most frequently documented among RSV (12.4%) and HMPV (11.7%) episodes. Diabetes mellitus was the most frequent pre-existing comorbidity among SARS-CoV-2 and Influenza A episodes (16.7% each).

### 3.7. Factors Associated with Length of Hospital Stay in Adults

Factors associated with length of hospital stay (LOS) among adult inpatients with confirmed viral respiratory episodes were examined using negative binomial regression on continuous LOS (nights) as the primary analysis, with modified Poisson regression for prolonged stay (>7 nights) as a sensitivity analysis (Table 6). Models used patient-clustered robust standard errors to account for repeated episodes.

In the fully adjusted primary model, older age was the strongest factor associated with longer hospital stays. Compared with patients aged 18–40 years, expected LOS was 45% longer at 41–59 years (IRR = 1.45, 95% CI: 1.29–1.64) and 54% longer at ≥60 years (IRR = 1.54, 95% CI: 1.38–1.72; both *p* < 0.001). Female sex was associated with a modest reduction in LOS (IRR = 0.91, 95% CI: 0.84–0.99, *p* = 0.025). These associations were directionally consistent in the sensitivity analysis (older-age RR = 1.73 and 1.89 for 41–59 and ≥60 years, respectively; both *p* < 0.001; female RR = 0.89, *p* = 0.006).

The specific virus detected during the encounter was associated with LOS. Compared with SARS-CoV-2, Influenza A (IRR = 0.82, 95% CI: 0.74–0.92, *p* = 0.001) and HMPV (IRR = 0.67, 95% CI: 0.55–0.80, *p* < 0.001) were independently associated with significantly shorter stays. In contrast, Influenza B (IRR = 0.88, 95% CI: 0.72–1.08, *p* = 0.222) and RSV (IRR = 1.09, 95% CI: 0.86–1.40, *p* = 0.472) did not differ significantly from SARS-CoV-2. Consistent with these findings, Influenza A and B had lower median LOS (5.0 nights) than SARS-CoV-2 (7.0 nights) and RSV (6.0 nights). In the sensitivity analysis, the Influenza B association reached statistical significance (RR = 0.76, 95% CI: 0.61–0.94, *p* = 0.013) while the HMPV estimate was borderline (RR = 0.67, 95% CI: 0.45–1.00, *p* = 0.050), indicating that these two effects were sensitive to model specification and warrant cautious interpretation.

Pre-existing comorbidities were independently associated with LOS. Heart failure and diabetes mellitus were associated with approximately 24% and 18% longer expected stays, respectively (IRR = 1.24, 95% CI: 1.08–1.43, *p* = 0.003; IRR = 1.18, 95% CI: 1.07–1.30, *p* = 0.001), reflected in higher median LOS (9.0 and 8.0 nights, respectively). Systemic hypertension (IRR = 1.09, 95% CI: 0.97–1.23, *p* = 0.155) and asthma (IRR = 0.89, 95% CI: 0.77–1.03, *p* = 0.129) were not significantly associated with LOS. Pulmonary hypertension (*n* = 23) was associated with substantially longer stays (median 13.0 nights) in both the primary (IRR = 2.03, 95% CI: 1.48–2.79, *p* < 0.001) and sensitivity (RR = 1.88, 95% CI: 1.42–2.47, *p* < 0.001) analyses; however, these estimates should be interpreted cautiously given the small number of patients with this comorbidity. A sensitivity analysis restricted to the post-restriction era (*n* = 1131) yielded materially identical results to the primary model. Influenza A (IRR = 0.63, 95% CI: 0.54–0.74, *p* < 0.001) and HMPV (IRR = 0.58, 95% CI: 0.46–0.73, *p* < 0.001) remained associated with significantly shorter stays compared to SARS-CoV-2, while RSV (IRR = 0.99, 95% CI: 0.77–1.27, *p* = 0.920) and Influenza B (IRR = 0.85, 95% CI: 0.62–1.17, *p* = 0.317) remained non-significant.

### 3.8. Length of Stay Analysis Among Pediatric Inpatients (<18 Years)

Length of stay (LOS) among pediatric inpatients (<18 years) with confirmed respiratory viral detection episodes was analyzed (Table 7). Descriptively, median LOS was similar across most viruses (4 nights for SARS-CoV-2, Influenza A, RSV, and Influenza B), with HMPV showing a slightly longer median of 5 nights (IQR 3–9).

While the global Kruskal–Wallis test indicated a statistically significant difference in LOS across pathogens (χ^2^ = 11.03, *p* = 0.026), Bonferroni-adjusted pairwise comparisons using Dunn’s post hoc test demonstrated that none of the virus-specific differences reached statistical significance (all adjusted *p* > 0.05). Consistently, in the multivariable negative binomial regression adjusted for age group and sex, no virus was independently associated with LOS (Influenza A: IRR = 0.90, 95% CI: 0.71–1.13, *p* = 0.347; RSV: IRR = 0.99, 95% CI: 0.86–1.15, *p* = 0.942; Influenza B: IRR = 1.03, 95% CI: 0.81–1.30, *p* = 0.814; HMPV: IRR = 1.19, 95% CI: 0.97–1.47, *p* = 0.099).

In contrast, age group was significantly associated with LOS. Compared to children aged 0–5 years, those aged 6–11 years had 29% longer expected stays (IRR = 1.29, 95% CI: 1.10–1.50, *p* = 0.001), and those aged 12–17 years had 56% longer expected stays (IRR = 1.56, 95% CI: 1.22–2.01, *p* < 0.001). Sex was not associated with LOS (IRR = 1.00, 95% CI: 0.89–1.12, *p* = 0.972).

## 4. Discussion

Acute respiratory infections remain a major global health burden, and their epidemiology varies by season, geographic location, and population characteristics. However, longitudinal surveillance data from Saudi Arabia remain limited, with available studies generally covering relatively short periods [15,16,17,18]. This study analyzed 5708 confirmed respiratory viral detection episodes caused by SARS-CoV-2, influenza A, influenza B, RSV, and HMPV between 2017 and 2025 at a major tertiary care center in Riyadh.

The current study showed that SARS-CoV-2 was the most frequently detected virus, accounting for 52.5% of confirmed viral detections. This predominance is expected, particularly during and after the COVID-19 pandemic periods. Adults aged ≥ 60 years and children aged 0–5 years represented the largest proportions of detected episodes, which is also consistent with previous reports showing children and older individuals as the most affected groups by respiratory pathogens [19,20]. In addition, children represented the majority of co-detection events, with 61.7% of all co-detected subjects aged 0–5 years. However, co-detections accounted for approximately 1.1% of all unique clinical episodes (60 of 5648), which was lower than the frequencies exceeding 10% reported in previous studies from the Riyadh region [18,21]. However, direct comparisons should be interpreted cautiously because of potential differences in study populations, testing strategies, and co-detection definition.

The current study showed that influenza A and SARS-CoV-2 were more frequently detected among adults and older adults, whereas RSV, HMPV, and influenza B were more common among children and younger individuals. This pattern is consistent with previous local and international studies reporting higher frequencies of RSV, HMPV, and influenza B in younger age groups, with a substantial burden of SARS-CoV-2 among adult populations [22,23,24,25,26].

The findings of the present study showed middle-aged and older adult inpatients were more likely to experience prolonged hospitalization than younger adults (18–40 years). Influenza A and HMPV detected during the encounter were associated with a modestly lower risk of prolonged LOS than SARS-CoV-2, consistent with previous studies reporting milder clinical outcomes with influenza A than with COVID-19 [27,28], whereas RSV and influenza B did not differ significantly from SARS-CoV-2. Among patients aged < 18 years, no virus, including HMPV, was independently associated with LOS after adjustment for age and sex; instead, older childhood age was associated with longer expected hospital stays.

The COVID-19 pandemic was associated with marked changes in the detection and seasonal patterns of major respiratory viruses. In the present study, the absolute number of detected respiratory viral episodes increased substantially during and after the post-restriction period; however, this finding should be interpreted with caution because the epidemiological eras differed in duration, and changes in testing volume could not be fully assessed [29,30,31]. In the pre-COVID-19 pandemic era, influenza A detection was dominant, followed by influenza B, with very few cases of RSV or HMPV. During the lockdown and restriction eras, there were very few observed detections of viruses other than SARS-CoV-2; influenza A and RSV were entirely absent during the lockdown era and remained rare through the restriction era. This pattern is consistent with global reports of substantially reduced observed detection of seasonal respiratory virus activity during 2020–2022 [31,32]. It has been suggested that the greater decline in detection of seasonal respiratory viruses, compared with SARS-CoV-2, may be partly attributable to pre-existing immunity, which could increase the exposure threshold required for infection with these viruses [32]. In addition, other studies suggested viral interference as an explanation for this decline, where infection with one virus results in an inhibitory effect on other viruses [30,33,34].

In the post-restriction era, RSV was the second most frequently detected virus after SARS-CoV-2, accounting for more than 28.3% of confirmed infections. Similar substantial increases in the detection of RSV and other respiratory viruses have been reported globally, particularly among children, following the easing of COVID-19 restrictions [35,36]. These rebounds have been attributed to reduced viral exposure during the pandemic and the resulting accumulation of susceptible individuals. Because RSV and HMPV predominantly affected younger patients in our cohort, their increased detection in the post-restriction period may partly explain the shift toward a younger age distribution, with patients aged <18 years accounting for the majority of detections in the post-restriction period. In contrast, older individuals represented the largest age group before the COVID-19 pandemic era.

The observed increases in the detection of RSV and influenza viruses in the post-restriction era, particularly among young children [35,36], underscores the importance of maintaining robust surveillance and prevention strategies. Our finding that older adults and patients with diabetes or heart failure are at increased risk of prolonged hospitalization [27] highlights groups that should be prioritized for clinical monitoring and early intervention. The winter predominance of RSV [37,38], alongside the seasonal clustering of influenza A in autumn and winter [39,40,41], supports the timing of seasonal prevention efforts. Continued molecular surveillance is essential to monitor shifts in pathogen circulation and to guide timely public health responses [29,30,31,32].

Seasonal shifts across the COVID-19 era could not be fully evaluated, particularly for RSV and HMPV, because of their limited number of pre-pandemic detections. Nevertheless, both viruses were detected predominantly during the winter season in the present study. This pattern is consistent with the local pre-COVID-19 era reports suggesting that their overall seasonality was preserved or re-established after the COVID-19 pandemic, despite temporary disruptions in circulation during the pandemic period [37,38]. Influenza A showed a predominantly autumn–winter pattern, whereas influenza B cases were concentrated mainly in winter. This pattern is broadly consistent with various post-pandemic surveillance studies, which show that influenza A infections generally peak earlier in the respiratory season, while influenza B tends to peak later [39,40,41].

In conclusion, this study is among the few long-term investigations to provide a longitudinal view of major respiratory viral pathogens detected in Saudi Arabia across the pre-COVID-19, pandemic, and post-restriction periods. Detections of non-SARS-CoV-2 respiratory viruses were markedly lower during the lockdown and restriction periods, followed by increased detection of RSV, HMPV, and influenza viruses, with clear age-specific and seasonal patterns. Older adults had a higher risk of prolonged hospitalization, whereas influenza A and HMPV were associated with a slightly lower risk than SARS-CoV-2. However, several limitations should be considered in the present study. The total number of tests performed during the study period was unavailable, limiting estimation of virus-specific positivity rates. Consequently, observed temporal changes in case counts may have been influenced by variations in testing volume, ordering practices, or patient selection over time, rather than true changes in pathogen circulation. This precludes direct comparison of detection rates across eras. Additionally, era-specific factors such as changes in admission criteria, bed pressure, and discharge practices during the pandemic may have confounded pathogen-LOS associations, and these could not be fully accounted for in the analysis. Adjustment for calendar era was attempted in the LOS models; however, because of the strong structural association between virus type and calendar era, and sparse virus-era combinations, stable simultaneous estimation could not be achieved. Admission and specimen-collection timestamps sufficient to compute the admission-to-test interval were unavailable. Therefore, infections present at admission could not be distinguished from those detected later during hospitalization; this introduces the risk of misattributing LOS to incidental or hospital-acquired detections. Furthermore, data on unmeasured confounders such as illness severity, ICU admission, oxygen/ventilatory support, immunocompromised status, and bacterial co-infection were unavailable. In addition, data for other common respiratory viruses, such as rhinovirus, enterovirus, and adenovirus, could not be extracted, potentially increasing the relative contribution of the included pathogens. Although patient-clustered robust standard errors were implemented to account for repeated episodes, and a sensitivity analysis restricted to one episode per patient yielded materially identical results, repeated observations from the same patient were possible and individual-level random effects could not be fully modeled. Furthermore, symptom and comorbidity data were extracted from structured electronic health record fields; because missing entries were not systematically documented, symptom prevalence may be underestimated, and we could not verify that all clinical elements were accurately recorded for every patient. This limits the precision of the comorbidity and symptom analyses. Finally, the retrospective single-center design may limit generalizability, although the study was conducted at a large tertiary care center serving a broad and diverse patient population. Multicenter surveillance studies using consistent testing strategies are needed in the future.

## Figures and Tables

**Table 1:** Demographic Characteristics of Confirmed Viral Respiratory Detection Episodes.

Characteristic	*n*	%
Total Viral Infection Episodes	5708	100%
Sex		
Male	2783	48.8%
Female	2925	51.2%
Age Group (years)		
0–5	1501	26.3%
6–17 years	719	12.6%
18–40 years	953	16.7%
41–59 years	924	16.2%
≥60 years	1611	28.2%
Nationality		
Saudi	4889	85.7%
Non-Saudi	819	14.3%
Non-Saudi nationalities (top 8)		
Yemeni	150	2.6%
Indian	101	1.8%
Syrian	87	1.5%
Sudanese	80	1.4%
Filipino	66	1.2%
Egyptian	65	1.1%
Jordanian	39	0.7%
Palestinian	34	0.6%
Other	197	3.5%
Confirmed Pathogen		
SARS-CoV-2	2996	52.5%
Influenza A	1090	19.1%
RSV	936	16.4%
Influenza B	481	8.4%
HMPV	205	3.6%
Care setting		
Inpatient	4518	79.2%
Non-inpatient	1167	20.4%
Unknown	23	0.4%

**Table 2:** Co-detection Patterns and Age Distribution Among Co-Detected Episodes.

Category	*n*	%
Pathogen Combination		
Influenza B + RSV	14	23.3%
SARS-CoV-2 + RSV	12	20.0%
Influenza A + RSV	10	16.7%
SARS-CoV-2 + Influenza A	8	13.3%
Influenza A + Influenza B	5	8.3%
HMPV + RSV	3	5.0%
SARS-CoV-2 + Influenza B	3	5.0%
HMPV + Influenza B	3	5.0%
SARS-CoV-2 + HMPV	2	3.3%
Age Group of Co-detected Cases		
0–5 years	37	61.7%
6–17 years	12	20.0%
18–40 years	3	5.0%
41–59 years	3	5.0%
≥60 years	5	8.3%

A viral detection episode was defined as a unique detection of a specific virus. To define the clinical episode operationally, we used a 14-day window: positive detections for any virus occurring within 14 days were considered part of the same clinical episode; detections occurring ≥14 days apart were counted as separate clinical episodes. The dataset comprised 5708 virus-specific detection episodes, representing 5648 unique clinical episodes across 5645 unique patients (3 patients experienced > 1 distinct episode). Because each co-detection event contributes two virus-specific episodes, the 60 co-detection events resulted in 120 virus-specific detections. Percentages in this table are calculated based on the 60 clinical episodes with co-detection. Age groups are mutually exclusive.

**Table 3:** Pathogen-specific Demographic Profile of Confirmed Viral Respiratory Detection Episodes.

			Sex (%)				Age Group Distribution (%)				
Virus	*n*	Age, Median (IQR)	Female%	Male%	Saudi%	Inpatient%	0–5	6–17	18–40	41–59	≥60
SARS-CoV-2	2996	47 (24–67)	51.5	48.5	81.2	81.9	15.1	7.0	20.1	21.3	36.5
Influenza A	1090	38 (9–65)	52.5	47.5	89.1	73.0	12.9	17.8	20.3	17.2	31.8
RSV	936	1 (0–6)	47.1	52.9	93.1	81.4	71.4	14.9	2.6	3.8	7.4
Influenza B	481	10 (4–39)	53.8	46.2	90.4	65.5	27.7	27.4	20.2	9.1	15.6
HMPV	205	4 (1–31)	54.1	45.9	86.8	93.7	51.7	22.0	4.4	8.8	13.2

**Table 4:** Age-group and respiratory virus distribution across COVID-19 eras.

Era (Duration)	Age Group Distribution *n* (%)					Respiratory Virus Distribution, *n* (Within-Era%)/Annualized Detection Frequency (Episodes/Year)				
	0–5	6–17	18–40	41–59	≥60	SARS-CoV-2	Influenza A	RSV	Influenza B	HMPV
Pre-COVID (29.6 mo)	87 (17.1%)	43 (8.4%)	112 (22.0%)	96 (18.9%)	171 (33.6%)	0 (0.0%)/0.0	362 (71.1%)/146.9	3 (0.6%)/1.2	132 (25.9%)/53.6	12 (2.4%)/4.9
Lockdown (3.0 mo)	5 (2.2%)	5 (2.2%)	45 (19.7%)	97 (42.4%)	77 (33.6%)	223 (97.4%)/885.4	0 (0.0%)/0.0	0 (0.0%)/0.0	2 (0.9%)/7.9	4 (1.7%)/15.9
Restriction (23.3 mo)	143 (8.2%)	114 (6.5%)	416 (23.8%)	398 (22.8%)	674 (38.6%)	1626 (93.2%)/837.7	71 (4.1%)/36.6	20 (1.1%)/10.3	11 (0.6%)/5.7	17 (1.0%)/8.8
Post-restriction (43.0 mo)	1266 (39.3%)	557 (17.3%)	380 (11.8%)	333 (10.3%)	689 (21.4%)	1147 (35.6%)/320.1	657 (20.4%)/183.3	913 (28.3%)/254.8	336 (10.4%)/93.8	172 (5.3%)/48.0

Annualized detection frequencies were calculated using exact era durations; era lengths in column headers are rounded to one decimal.

**Table 5:** Presenting symptoms, respiratory diagnoses, and chronic comorbidities by respiratory virus.

Comorbidity/Symptom	Overall (*n* = 5708)	SARS-CoV-2 (*n* = 2996)	Influenza A (*n* = 1090)	RSV (*n* = 936)	Influenza B (*n* = 481)	HMPV (*n* = 205)
Presenting Symptoms						
Fever	1867 (32.7%)	704 (23.5%)	382 (35.0%)	451 (48.2%)	228 (47.4%)	102 (49.8%)
Cough	744 (13.0%)	216 (7.2%)	132 (12.1%)	291 (31.1%)	60 (12.5%)	45 (22.0%)
Shortness of breath	891 (15.6%)	600 (20.0%)	171 (15.7%)	49 (5.2%)	49 (10.2%)	22 (10.7%)
Respiratory Diagnoses						
Pneumonia, viral/CAP ^1^	391 (6.9%)	267 (8.9%)	61 (5.6%)	28 (3.0%)	26 (5.4%)	9 (4.4%)
Pneumonia, any ^2^	1099 (19.3%)	600 (20.0%)	257 (23.6%)	117 (12.5%)	79 (16.4%)	46 (22.4%)
Bronchiolitis	279 (4.9%)	20 (0.7%)	10 (0.9%)	225 (24.0%)	7 (1.5%)	17 (8.3%)
Chronic Comorbidities						
Diabetes mellitus	752 (13.2%)	499 (16.7%)	182 (16.7%)	23 (2.5%)	40 (8.3%)	8 (3.9%)
Hypertension ^3^	510 (8.9%)	331 (11.0%)	125 (11.5%)	17 (1.8%)	29 (6.0%)	8 (3.9%)
Pulmonary hypertension	25 (0.4%)	10 (0.3%)	9 (0.8%)	1 (0.1%)	3 (0.6%)	2 (1.0%)
Heart failure	210 (3.7%)	111 (3.7%)	64 (5.9%)	12 (1.3%)	18 (3.7%)	5 (2.4%)
Asthma	390 (6.8%)	105 (3.5%)	108 (9.9%)	116 (12.4%)	37 (7.7%)	24 (11.7%)

^1^ Viral or community-acquired pneumonia (EHR-recorded clinical diagnosis; not independently adjudicated for etiologic attribution). ^2^ Any pneumonia diagnosis, including hospital-acquired and bacterial. ^3^ Systemic hypertension; pulmonary hypertension reported separately. Categories are not mutually exclusive. Complications were unreported in 23 episodes (0.4%).

**Table 6:** Factors associated with length of hospital stay among adult inpatients with confirmed respiratory viral detection episodes.

Variable	Median LOS, Nights (IQR)	Episodes, *n*	Primary—Negative Binomial (LOS in Nights)		Sensitivity—Modified Poisson (Prolonged > 7 Nights)	
			IRR (95% CI)	*p*	RR (95% CI)	*p*
Age group						
18–40 (ref)	4.0 (2.0–7.0)	710	1.00 (ref)	-	1.00 (ref)	-
41–59 years	7.0 (3.0–12.0)	768	1.45 (1.29–1.64)	<0.001	1.73 (1.48–2.01)	<0.001
≥60 years	8.0 (4.0–14.0)	1431	1.54 (1.38–1.72)	<0.001	1.89 (1.64–2.18)	<0.001
Sex						
Male (ref)	7.0 (3.0–13.0)	1338	1.00 (ref)	-	1.00 (ref)	-
Female	6.0 (3.0–11.0)	1571	0.91 (0.84–0.99)	0.025	0.89 (0.82–0.97)	0.006
Virus						
SARS-CoV-2 (ref)	7.0 (3.0–13.0)	2011	1.00 (ref)	-	1.00 (ref)	-
Influenza A	5.0 (3.0–10.0)	586	0.82 (0.74–0.92)	0.001	0.75 (0.67–0.85)	<0.001
RSV	6.0 (3.0–12.2)	112	1.09 (0.86–1.40)	0.472	0.89 (0.70–1.12)	0.321
Influenza B	5.0 (3.0–9.0)	150	0.88 (0.72–1.08)	0.222	0.76 (0.61–0.94)	0.013
HMPV	6.0 (4.0–9.8)	50	0.67 (0.55–0.80)	<0.001	0.67 (0.45–1.00)	0.050
Comorbidities (ref: absent)						
Diabetes mellitus	8.0 (4.0–15.0)	710	1.18 (1.07–1.30)	0.001	1.21 (1.10–1.32)	<0.001
Hypertension	8.0 (4.0–15.0)	490	1.09 (0.97–1.23)	0.155	1.01 (0.91–1.13)	0.803
Pulmonary hypertension	13.0 (8.5–24.0)	23	2.03 (1.48–2.79)	<0.001	1.88 (1.42–2.47)	<0.001
Heart failure	9.0 (5.0–17.0)	206	1.24 (1.08–1.43)	0.003	1.16 (1.02–1.33)	0.028
Asthma	7.0 (4.0–12.8)	154	0.89 (0.77–1.03)	0.129	1.07 (0.90–1.28)	0.417

IRR, incidence rate ratio; RR, risk ratio; IQR, interquartile range; LOS, length of stay. Of 2962 adult single-virus inpatient episodes, 53 were excluded for missing length-of-stay data or missing covariates, leaving 2909 adults (2908 unique patients). Models used patient-clustered robust standard errors to account for repeated episodes (dispersion parameter alpha = 0.68). A sensitivity analysis restricted to one randomly selected episode per patient (*n* = 2908) yielded materially identical results. Hypertension in this table refers to systemic hypertension. Associations reflect the virus detected during the encounter rather than established causation, as admission-to-test interval timestamps were unavailable.

**Table 7:** Factors associated with length of hospital stay among pediatric inpatients (<18 years) with confirmed respiratory viral detection episodes.

Variable	Median LOS, Nights (IQR)	Episodes, *n*	IRR (95% CI)	*p*
Virus				
SARS-CoV-2 (ref)	4.0 (2.0–7.0)	397	1.00 (ref)	-
Influenza A	4.0 (2.0–6.0)	166	0.90 (0.71–1.13)	0.347
RSV	4.0 (3.0–7.0)	598	0.99 (0.86–1.15)	0.942
Influenza B	4.0 (3.0–8.0)	140	1.03 (0.81–1.30)	0.814
HMPV	5.0 (3.0–9.0)	134	1.19 (0.97–1.47)	0.099
Age group				
0–5 (ref)	4.0 (3.0–7.0)	1026	1.00 (ref)	-
6–11	4.0 (3.0–8.0)	276	1.29 (1.10–1.50)	0.001
12–17	5.0 (2.0–10.0)	133	1.56 (1.22–2.01)	<0.001
Sex				
Male (ref)	4.0 (3.0–7.0)	774	1.00 (ref)	-
Female	4.0 (3.0–7.0)	661	1.00 (0.89–1.12)	0.972

IRR, incidence rate ratio; CI, confidence interval; IQR, interquartile range; LOS, length of stay; ref, reference category. Of 1454 pediatric single-virus inpatient episodes, 19 were excluded for missing length-of-stay data or missing covariates, leaving 1435 children (1434 unique patients). Models used patient-clustered robust standard errors to account for repeated episodes (dispersion α = 0.53). A sensitivity analysis restricted to one randomly selected episode per patient (*n* = 1434) yielded materially identical results.

## Data Availability

The original contributions presented in this study are included in the article/Supplementary Materials. Further inquiries can be directed to the corresponding author.

## Supplementary Materials

The following supporting information can be downloaded at: https://www.mdpi.com/article/10.3390/pathogens15090918/s1, Figure S1. Flow diagram of study population selection and length-of-stay analysis cohorts.
